# Extraordinary first jejunal arterial variation associated with annular pancreas undergoing pancreaticoduodenectomy for pancreatic cancer: a case report

**DOI:** 10.1007/s00276-020-02671-9

**Published:** 2021-01-22

**Authors:** Yasuhito Iwao, Daisuke Ban, Satoru Muro, Atsushi Kudo, Shinji Tanaka, Krishna Menon, Minoru Tanabe

**Affiliations:** 1 Department of Hepatobiliary and Pancreatic Surgery, Graduate School of Medical and Dental Science, Tokyo Medical and Dental University, 1-5-45 Yushima, Bunkyo-ku, Tokyo, Japan; 2 Department of Clinical Anatomy, Graduate School of Medical and Dental Science, Tokyo Medical and Dental University, 1-5-45 Yushima, Bunkyo-ku, Tokyo, Japan; 3 Molecular Oncology, Graduate School of Medical and Dental Science, Tokyo Medical and Dental University, 1-5-45 Yushima, Bunkyo-ku, Tokyo, Japan; 4 Department of Hepatobiliary and Pancreatic Surgery, Institute of Liver Studies, King’s College Hospital, Denmark Hill, London, UK

**Keywords:** Jejunal artery, Annular pancreas, Arterial variation, Anatomy, Embryology

## Abstract

**Purpose:**

Annular pancreas encountered in adults and jejunal arterial variations are rare. Anatomical variations can cause conflicts between oncology and surgical safety.

**Methods:**

Case report of a 68-year-old man suffering from vomiting because of an annular pancreas and a ductal adenocarcinoma of the pancreas head invading the second portion of the duodenum.

**Results:**

Contrast-enhanced computed tomography showed multiple arterial variations describing the absence of the coeliac trunk such that the left gastric artery (LGA), splenic artery and superior mesenteric artery (SMA) were arising separately from the aorta. The accessory left hepatic artery arose from the LGA; and both the common hepatic artery and combined trunk of the replaced right hepatic artery with the higher replaced first jejunal artery separately arose close to the root of the SMA. The patient underwent curative pancreaticoduodenectomy which achieved 3 years of recurrence-free survival.

**Conclusion:**

This was an extraordinary case of annular pancreas with first jejunal arterial variation detailing an embryological interpretation as well as considerations for balancing short- and long-term outcomes.

## Introduction

Annular pancreas is a rare congenital malformation in adults [13, 16, 17], although the anomaly is well described in paediatrics. It usually presents with a duodenal obstruction at the first day of life, requiring surgical intervention. The incidence of annular pancreas is 1:20,000 new births [9, 13]. Conversely, the prevalence of annular pancreas reported in adults varies widely between 1:20,000 from an autopsy series [15, 16] and 1:250 from an endoscopic study [5, 16]. Most cases of annular pancreas in adults have been found accidentally, accompanying other lesions or diseases causing symptoms. Therefore, annular pancreas is regarded as a risk factor of neoplasms [16, 17]. The combination of annular pancreas and arterial variations has been rarely reported. In fact, a small study concluded no correlation between anomalous pancreas formation and variations of hepatic arteries [11].

Oncological benefit and safety of treatment can come at odds because of anatomical variations. For instance, a replaced right hepatic artery (repRHA) arising from the superior mesenteric artery (SMA) during pancreaticoduodenectomy (PD), also known as Whipple’s procedure, is an obviously difficult surgery in terms of satisfactory achievement for both survival benefit and surgical safety [2, 7]. Pancreatic ductal adenocarcinoma (PDAC) is well known to preferentially invade microvascular structures causing poor outcomes and remains challenging to evaluate preoperatively, even with the most advanced radiological modalities. As such, PD for PDAC becomes far more complicated in cases involving anatomical variations.

Variations of the coeliac trunk (CoT) and the SMA have been described in depth by Adachi and Michels [1, 10]. CoT variations have also been interpreted in the context of the embryological development of abdominal organs [6]. However, jejunal arterial variations are extremely rare and may be excluded from this model of development. Following an intensive search, we found only two previous studies describing a variant jejunal artery arising from the CoT [12, 14]. In this article, we present an extraordinary case of a complex arterial anatomy associated with annular pancreas undergoing PD for PDAC.

## Case report

**Fig. 1:**
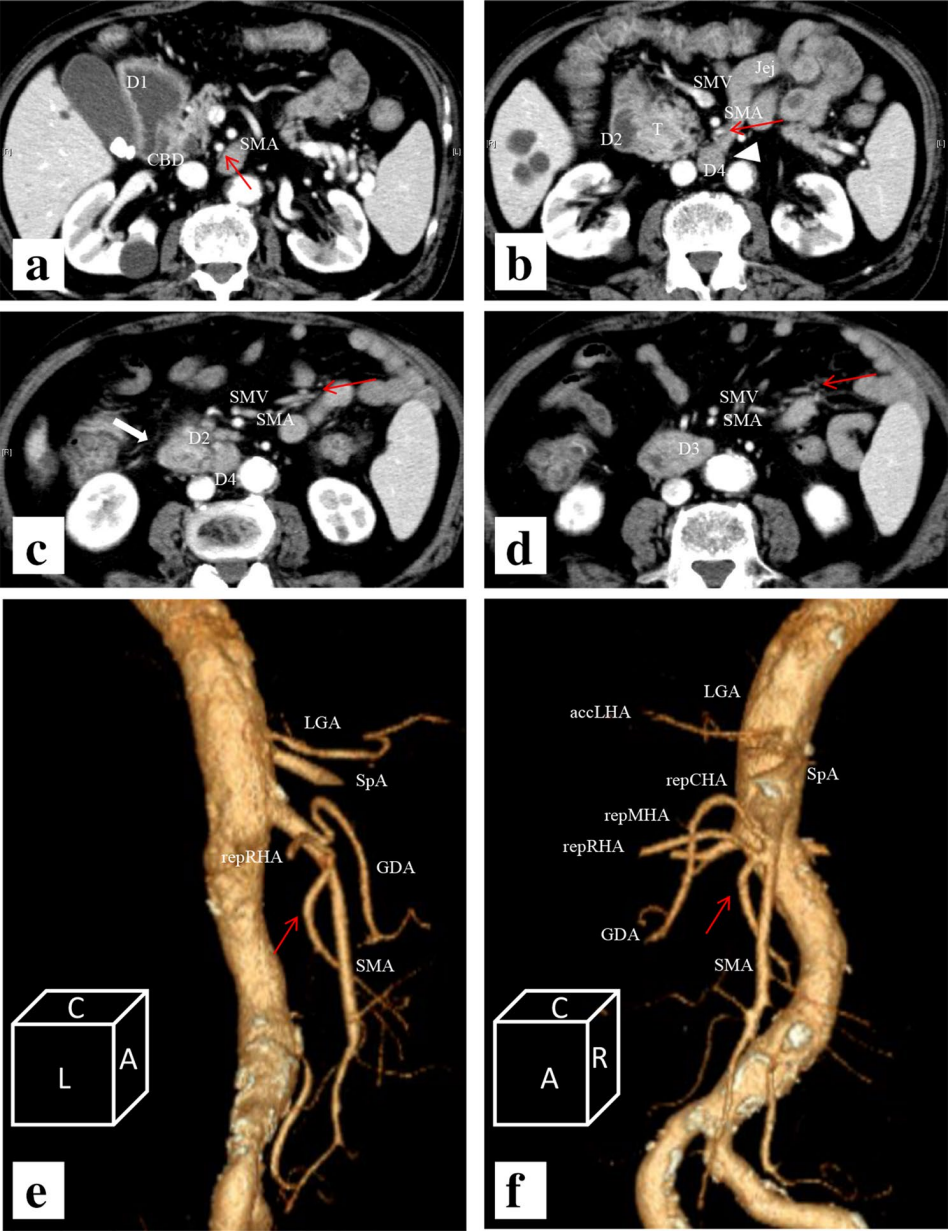
Contrast-enhanced computed tomography (CT) **a** showed a dilated first portion of the duodenum and an intact intra-pancreatic common bile duct. Both the higher replaced first jejunal artery (repFJA) (red arrow) and superior mesenteric artery (SMA) were similar in diameter, i.e. 7 and 9 mm, respectively, thereby, resembling duplicated SMAs. **b** Low-density lesion invading the second portion of the duodenum (D2), but not involving the repFJA (red arrow), SMA or superior mesenteric vein (SMV). In addition, the transposition of the fourth portion of the duodenum (D4) and jejunum did not reach the left side of the aorta, but stopped at the right side of it (arrowhead). **c** CT demonstrated D2, D4, repFJA (red arrow), SMA and SMV. Soft-density tissue (thick arrow) surrounding D2 might be the part of annular pancreas located at the caudal side of the pancreatic head tumour. **d** CT displayed repFJA (red arrow), SMA, SMV and a very short third portion of the duodenum. **e** Reconstructed CT angiography in the left lateral position depicted three arteries arising from the aorta namely, the left gastric artery (LGA) bearing the accessory left hepatic artery (accLHA), splenic artery (SpA) and SMA. The replaced common hepatic artery (repCHA), from which the gastroduodenal artery (GDA) and replaced middle hepatic artery (repMHA) arose, and a common trunk of the replaced right hepatic artery (repRHA) and higher repFJA (red arrow) were, respectively, bifurcated near the root of the SMA. **f** Reconstructed CT angiography in the right anterior oblique added for the comprehension of the complicated arterial variations as described. *AccLHA* accessory left hepatic artery, *CBD* common bile duct, *D1* first portion of the duodenum, *D2* second portion of the duodenum, *D3* third portion of the duodenum, *D4* fourth portion of the duodenum, *GDA* gastroduodenal artery, *Jej* jejunum, *LGA* left gastric artery, *PV* portal vein, *repCHA* replaced common hepatic artery, *repMHA* replaced middle hepatic artery, *repRHA* replaced right hepatic artery, *SpA* splenic artery, *SMA* superior mesenteric artery, *SMV* superior mesenteric vein, *T* tumour

**Fig. 2:**
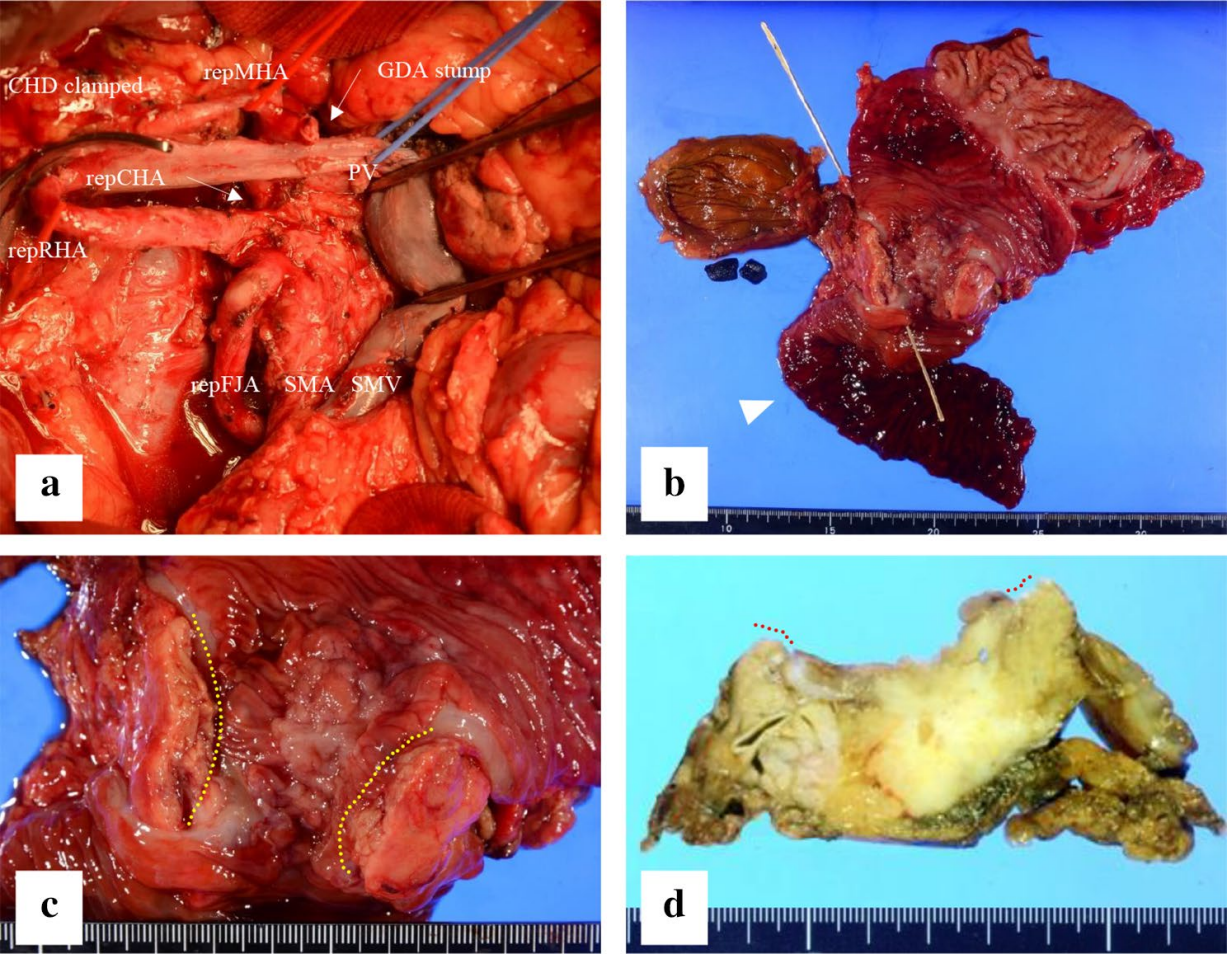
Intra-operative photograph showing **a** the complex arterial variations were identically preserved as pre-operative imaging, except for the gastroduodenal artery. After consideration of both the distance between the tumour and vessels and the patient’s medical condition, which required a combination of immunosuppressors, most of the nerve plexus surrounding arteries except for the superior mesenteric artery (SMA) trunk were resected to achieve maximal oncological benefit and surgical safety. Due to this surgical management, the diameter of the replaced first jejunal artery seemed to be apparently smaller than that of the SMA. **b** In the fresh specimen, the first portion of the duodenum appeared erosive and dilated and the second portion of the duodenum showed tumour invasion encircled by annular pancreas at the oral side of the ampulla of Vater. The ampulla was located by the metallic instrument inserted from the cut end of the common hepatic duct. Arrowhead indicates the duodenojejunal flexure; the lengths of the third and fourth portions of the duodenum in this specimen appear shortened. **c** The dotted line depicts the incision on the annular pancreas in the fresh specimen. **d** Formalin-fixed specimen showing invasion of tumour limited to a hemicircular portion of the duodenum, but in combination with annular pancreas led to vomiting in the patient. *CHD* common hepatic duct, *GDA* gastroduodenal artery, *PV* portal vein, *repCHA* replaced common hepatic artery, *repFJA* replaced first jejunal artery, *repMHA* replaced middle hepatic artery, *repRHA* replaced right hepatic artery, *SMA* superior mesenteric artery, *SMV* superior mesenteric vein

The patient was a 68-year-old man suffering from vomiting without jaundice. He had a medical history of liver cirrhosis related to autoimmune hepatitis treated and then maintained by 7 mg of predonine daily for the past 7 years. He was then diagnosed with Hashimoto disease. He experienced repeated epicarditis that required epicardial drainage and developed rheumatoid arthritis sustained by methotrexate (8 mg/day). While blood examination revealed normal liver function regulated by steroid maintenance, elevated tumour markers revealed a carcinoembryonic antigen (CEA) of 59.7 ng/mL and carbohydrate antigen 19-9 (CA19-9) concentration of 8932 units/mL. Oesophagogastroduodenoscopy (OGD) showed an almost obstructed stenosis at the second portion of the duodenum. Contrast-enhanced computed tomography (CT) images revealed a PDAC 35 mm in diameter in the pancreas head invading the duodenum, as well as a complex of multiple arterial variations without any distant metastases (Fig. 1a–d). The CT findings were confirmed by biopsy. This combination of variations involved the absence of CoT such that the left gastric artery (LGA), splenic artery (SpA) and SMA were separately arising from the aorta. The accessory left hepatic artery (accLHA) arose from the LGA; and both the common hepatic artery (CHA) and a combined trunk of the replaced right hepatic artery (repRHA) with the higher replaced first jejunal artery (repFJA) arose close to the root of the SMA (Fig. 1e, f). After assessment during resectable PDAC, the patient underwent subtotal stomach-preserving PD with all variated arteries preserved (Fig. 2a). At kocherisation, it was realised that the duodenum was short (approximately eight fingers-widths in length) and was mobilised more easily. Due to this curative resection, the patient has achieved 3-year recurrence-free survival to date. Specimen pathology was diagnosed as an infiltrative PDAC in the pancreas head invading directly to the duodenum with some positive lymph nodes as T2N1M0 stage IIB, based on the Eighth Edition of the American Joint Committee on Cancer and the Union for International Cancer Control Staging Manual [8].

## Discussion

Considering the inherent conflict between the biological behaviour of PDAC and the series of arterial variations observed in this case, it could have been optional to resect the variant jejunal artery for oncological purpose. However, because this variant jejunal artery fed a considerable section of the jejunum, given its significant diameter (Figs. 1a, 2a), and also maintained a distance from the tumour (Fig. 1a, b), this arterial variation had to be preserved. Despite a complicated dissection of these uncommon blood vessel variations in surgery, we achieved a margin-negative resection and an acceptable outcome, due to proper pre-operative radiological assessment and intra-operative surgical management.

In this combination of arterial variations, the LGA and SpA did not form a common trunk such as that found in Adachi Type V vascular variation (reported in 0.4% of cases) [1], but it can be identified because the CHA did not fuse with them, similar to a Michels Type IX classification, which is reported in 4.5% of cases [10]. In contrast, it was unusual that the CHA and repRHA did not accompany each other despite arising from the same trunk of the SMA. In other words, this case was completely different from the well-described hepatic arterial variations classified as Michels Type IX, in which the replaced CHA arises from the SMA. Furthermore, the repFJA joined the repRHA instead of the main trunk of the SMA. Both arterial findings are extremely rare.

According to the current theory of embryological development, at the sixth week of gestation, the pancreas starts developing. The ventral bud of the pancreas starts rotating to the right (clockwise) to be carried dorsally along with the common bile duct. Incomplete movement of the ventral bud of the pancreas leaves a remnant ring of tissue, forming the basis of annular pancreas [17]. In the following week of gestation, the ventral bud fuses with the dorsal bud. The stomach and duodenum including the fused pancreas then rotates. The spleen undergoes both reduction and relocation of the visceral vessels [6]. Consequently, there is a significant variability in the blood supply to the gut and the liver, which has been well described as variations of the CoT and SMA [1, 10].

**Fig. 3:**
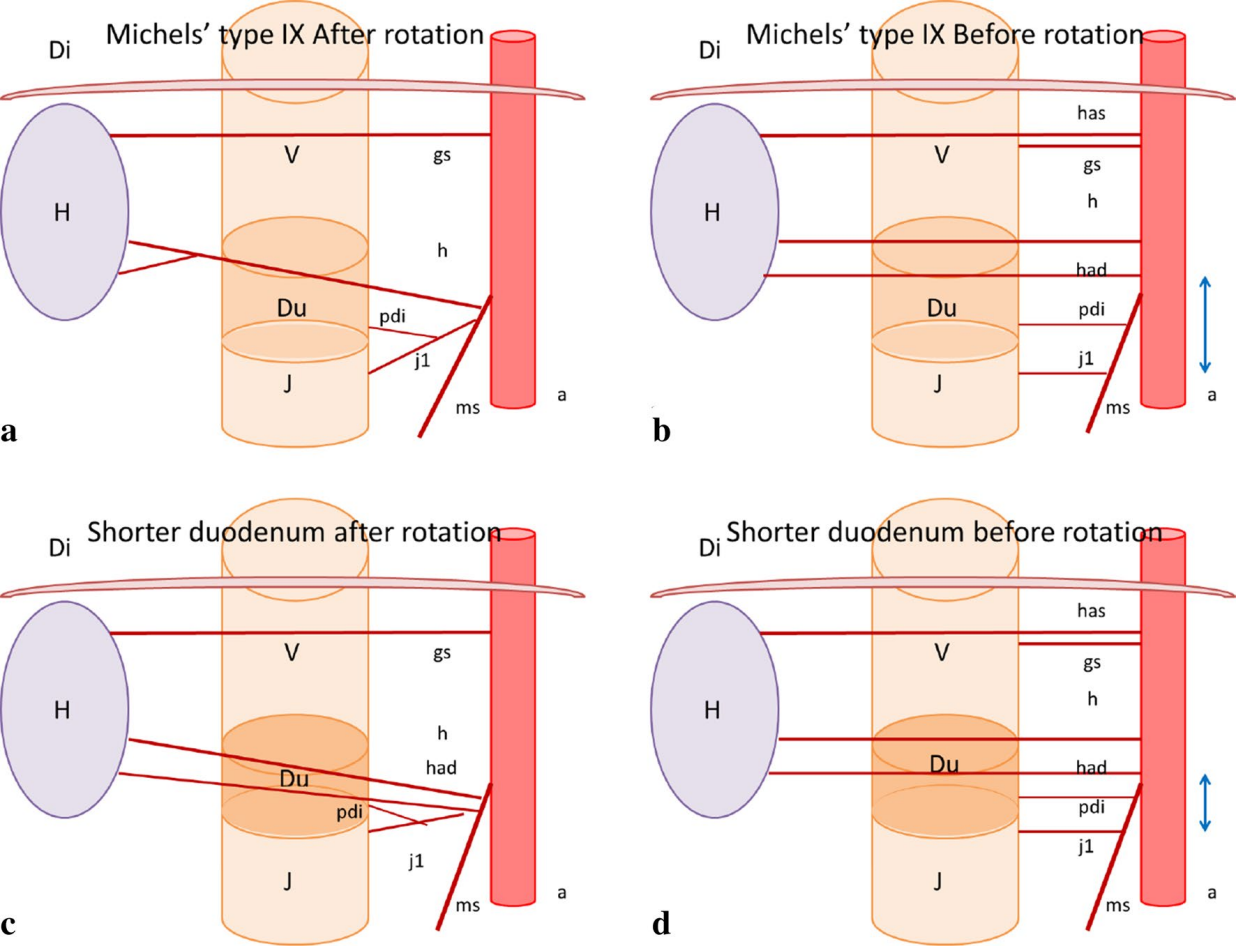
Schematics describing our embryological interpretation of arterial variations and duodenal anomaly. **a** Schematic of the type IX arterial variation in the Michel’s classification. This type was described as a combination of the accessory left hepatic artery arising from the left gastric artery and the complete replaced common hepatic artery arising from the superior mesenteric artery. **b** Schematic of the Michel’s Type IX before the rotation of the gut completed in the 10th week of gestation. The blue bilateral arrow indicates the distance between the foetal right hepatic artery and first jejunal artery. **c** Schematic of the current case of the short duodenum. **d** Schematic of this case before the rotation. The shorter blue bilateral arrow is a possible interpretation of the complex combination of arterial variations found in our patient. *A* aorta, *Di* diaphragma, *Du* duodenum, *gs* arteria gastrica sinistra, *H* hepatia, *h* arteria hepatica, *had* arteria hepatica accessoria dextra, *has* arteria hepatica accessoria sinistra, *J* jejunum, *j1* arteria jejunale prima, *pdi* arteria pancreaticoduodenalis inferior, *V* ventri

In our patient, we found that there were also pancreatic and duodenal anomalies (Fig. 2b–d). Annular pancreas is not particularly associated with arterial variants [11], but is more likely to be combined with duodenal malformations [13, 17]. In contrast, Akita argued that in the context of the embryological interpretation for a very rare common trunk between the splenic artery and SMA, intra-pancreatic arterial variations may be strongly associated with aberrant arterial formation on the CoT and SMA [3]. Annular pancreas can be considered as a consequence of the hypoplasty and irregular movement of the ventral bud of the pancreas [4], which may have an anomalous intra-pancreatic arterial arcade. On this basis, this deformation of the pancreas is also associated with a shorter duodenum particularly after the ampulla of Vater, as shown in Fig. 2b. The distal duodenum is expected to pass in front of the aorta to its left side. However, in the present case, it stopped just to the right of the aorta (Fig. 1b, d). Therefore, we speculate that this unusual duodenal morphology contributed to the formation of at least an independent branch consisting of the repRHA and repFJA as well as avoidance of the repRHA joining in the CHA, which in turn originates from the SMA, and the repFJA fusing with the main trunk of the SMA. As shown in Fig. 3a–d, the length of the anal half of the duodenum may have affected the distance between the repRHA and FJA during embryonic development, before rotation of the gut completed at gestational week 10 (Fig. 3b, d). Two previous reports of a variant of the FJA did not provide an embryological interpretation of their own cases [12, 14]. Although difficult to ascertain because of its rarity, a variant of the FJA may accurately reflect the etymology of the duodenum, which in the intestine is 12 finger-widths in length. However, it was not presented in this case.

## Conclusion

To conclude, this was an extremely rare case of multiple arterial variations, notably, a variant jejunal artery associated with annular pancreas and pronouncedly short duodenum, which has allowed us an opportunity to present an embryological interpretation into such occurrences. Furthermore, this case perfectly illustrates the sometimes confounding conflict between oncological benefit and surgical safety in the face of anatomical variations, as well as possible unprecedented considerations in balancing short- and long-term outcomes.

## Data Availability

The authors are accountable for all aspects of the work in ensuring that questions related to the accuracy or integrity of any part of the work are appropriately investigated and resolved.
